# Quality assurance of registration of CT and MRI data sets for treatment planning of radiotherapy for head and neck cancers

**DOI:** 10.1120/jacmp.v5i1.1951

**Published:** 2004-05-25

**Authors:** Craig S. Moore, Gary P. Liney, Andrew W. Beavis

**Affiliations:** ^1^ Department of Medical Physics, Hull and East Yorkshire NHS Trust and Department of Academic Medical Physics University of Hull Princess Royal Hospital Saltshouse Road Kingston Upon Hull HU8 9HE United Kingdom; ^2^ Centre for Magnetic Resonance Imaging University of Hull Hull Royal Infirmary Kingston Upon Hull HU3 2JZ United Kingdom

**Keywords:** radiotherapy, treatment planning, multimodality image registration

## Abstract

We are implementing the use of magnetic resonance (MR) images for head and neck radiotherapy planning, which involves their registration with computed tomography (CT). The quality assurance (QA) of the registration process was an initial step of this program.

A phantom was built, and appropriate materials were identified to produce clinically relevant MR $T_{1}$ and $T_{2}$ contrast for its constituent “anatomy.” We performed a characterization of the distortion detectable within our phantom. Finally, we assessed the accuracy of image registration by contouring structures in the registered/fused data sets using the treatment planning system. Each structure was contoured using each modality, in turn, blind of the other. The position, area, and perimeter of each structure were assessed as a measure of accuracy of the entire image registration process.

Distortion effects in the MR image were shown to be minimized by choosing a suitable (≥±30 kHz) receiver bandwidth. Remaining distortion was deemed clinically acceptable within ±15 cm of the magnetic field isocenter. A coefficient of agreement (*A*) analysis gave values to be within 9% of unity, where

$$A=\sqrt{R_{a}R_{p}}\;\text{and}\;R_{a/p}$$

is the ratio of the area/perimeter of a particular structure on CT to that on MR. The center of each structure of interest agreed to within 1.8 mm.

A QA process has been developed to assess the accuracy of using multimodality image registration in the planning of radiotherapy for the head and neck; we believe its introduction is feasible and safe.

PACS numbers: 87.53.Xd, 87.57.Gg, 87.59.Fm; 87.61.‐c, 87.66.Xa

## I. INTRODUCTION

Modern three‐dimensional radiotherapy treatment planning of cancer demands we use volumetric image data sets to design the conformal therapy of tumors, and conformal avoidance of the proximal, dose limiting, organs at risk.^(1)^ Most commonly, X ray computed tomography (CT) is used to provide this three‐dimensional model of the patient; furthermore, the Hounsfield/CT number information it provides can be calibrated to give an electron density map of the patient, which is used to make corrections in dose calculations. In order to improve the accuracy of the treatment modeling, there are two aspects of the calculations to consider: One is choosing the correct algorithm for the specific application^(2)^; the other is accurately describing the geometry of the tumor and surrounding tissues. The first point can be addressed by choosing a convolution model calculation.^(3)^ Fast Fourier Transform (FFT)‐based algorithms can be used for most anatomical sites^(4)^ and “full convolution” superposition^(5)^ based algorithms when density corrections play a vital role, for example, in the lung. This point reinforces the need for X ray CT descriptions of the patient, which give electron density information. The second point can be addressed by including imaging data from other modalities, which provide better soft tissue delineation (magnetic resonance imaging, MRI) or functional imaging (MR spectroscopy, functional MRI, positron emission tomography). Magnetic resonance imaging is now widely available to oncology departments, and it is beginning to become a popular option for treatment planning of radiotherapy. It has been suggested that MRI is the modality of choice^(6)^ for head and neck diagnostic imaging. Prior to the implementation of our current treatment planning system (CMS FOCUS/ XiO), we implemented planning of brain tumors using MRI alone.^(7)^ Our current planning system now has the ability to input imaging data from different modalities. These can be registered using the FOCALFusion (CMS) software. We are, therefore, implementing multimodality planning for anatomical sites where enhanced tumor/organ delineation and more sophisticated dose calculations will provide benefit.

The work in this manuscript relates to the implementation of the inclusion of MRI to our conformal (CT‐based) head and neck planning. Computed tomography data is obtained on our dedicated PQ5000 CT scanner (Philips) and the MRI images from a 1.5 T Signa EchoSpeed MR scanner (GE) in radiology. Once in the planning system, the two image sets are registered using FOCAL Fusion from CMS. Following this, they are used interchangeably to contour structures. It is well known that MR images are prone to distortion, which can introduce uncertainties in their geometrical accuracy. Distortions arise from both system‐ and patient‐induced effects; the former are due to inhomogeneities in both the main magnetic field and linear imaging gradients, while the latter is due to changes in magnetic susceptibility and chemical shift effects. It has been shown that distortion is inversely proportional to gradient strength.^(7)^ ^,^ ^(8)^ Furthermore, system‐induced distortions are enhanced at the periphery of the field and are expected to be minimized over a certain imaging volume centered on the isocenter of the magnet.^(9)^ Depending on the magnitude and extent of these distortions, image correction may or may not be considered necessary. Whereas the current literature contains material on correction of gradient‐induced distortions and other image artifacts, we feel that the particular aspects of the use of MRI in radiotherapy planning have not been addressed. In particular, we wished to demonstrate a systematic analysis that provides quantitative information on the accuracy of the registration process. To do this, we utilized a phantom that is considered clinically relevant, in that its design should reflect the anatomy under study.

Therefore, a relevant question to be considered is the following: How accurate and reliable is the tumor volume depicted in the secondary (in this case MRI) data set? A second question to answer is: how well do automatic registration processes perform, such as the Mutual Information algorithm^(10)^ ^,^ ^(11)^ implemented in our planning system? In order to investigate these issues, we designed and performed a series of experiments that are presented in this paper.

## II. METHODS AND MATERIALS

**Figure 1:**
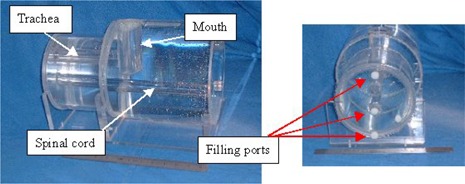
Two views of the phantom constructed and used in this work. Note the access ports that enable the various features of the phantom to be filled/emptied with fluids appropriate to the scanning modality and sequence used in each instance.

A head and neck phantom (Fig. 1) was built using a design similar to that reported by Meyer et al.^(12)^ We investigated the most appropriate materials to fill the phantom for the types of imaging sequences that would be used.

At our radiotherapy center, a “head jig” immobilization technique is used, which negates the possibility of using the radiofrequency (RF) head coil, which, under normal radiology conditions produces optimal head and neck images. Although our standard head and body coils are of the optimized “birdcage” design,^(13)^ the increased size of the body coil indicates the signal‐to‐noise ratio (SNR) and image homogeneity are both worse with this coil. As in our previous work,^(7)^ we propose using the body coil to obtain head and neck radiotherapy planning scans. Images of the phantom were obtained in the two coils to provide some quantitative analysis of using the seemingly less optimal configuration.

Given this choice of using the body coil, a scanning protocol that was expected to be useful was selected. We felt it prudent, therefore, to assess the magnetic field distortion and chemical shift effects.

Finally, we address the question central to this work: assessment of the validity and accuracy of the registration process and its reliability for radiotherapy planning for head and neck cases.

### A. Head and Neck Phantom Design and Filling Materials

The phantom was constructed from MR‐compatible materials (Perspex). Essentially, the phantom had to be constructed from materials that do not have undesirable properties, such as RF absorption, and greatly differing susceptibilities between different parts of the phantom. The susceptibility difference between water and Perspex is only 0.004 ppm, which will produce very little distortion. Perspex is also nonconductive and has an RF absorption similar to that of soft tissue.

The dimensions and filling liquids of the phantom were chosen to reflect *in vivo* characteristics. The phantom consisted of four compartments, which included a trachea and mouth (open to atmosphere), a spinal cord to simulate cerebrospinal fluid, a homogeneous brain and neck region, and a compartment to mimic subcutaneous fat in the neck.

Optimal phantom filling materials were then investigated. The most common materials used in phantom construction to date have included paramagnetic solutions, agar gels, and oils.^(14)^ ^,^ ^(15)^ To determine the required MR contrast properties for each compartment of the phantom, three volunteers were imaged. The volunteers were scanned using a clinically relevant $T_{1}$ weighted spin echo sequence [TR/TE=360/20 ms, slice thickness/gap=5/205 mm, image matrix=256×128, field‐of‐view (FOV)=25×25 cm, and 2 averages (NEX)] and a clinically relevant $T_{2}$ weighted spin echo sequence (TR/TE=3000/120, image matrix=256×256, FOV=25×25 cm, and 2 NEX). A reference gel (Diagnostic Sonar Ltd, Livingston) contained in a test tube was placed behind the neck and imaged simultaneously.

Region‐of‐interest (ROI) analysis was then carried out to determine mean signal intensities from fat, brain, CSF, and the reference gel. The ROIs were positioned close to the gel to obtain a similar $B_{1}$ profile of the head coil. Region‐of‐interest sizes were approximately 140 mm^2^ in the gel and brain but smaller within CSF and fat (80 mm^2^). Ratios of the signal intensity of fat, brain, and CSF to that of the reference gel for both sequences were determined.

Various materials were examined for their similarity to MR contrast properties *in vivo*. A collection of 500 mL container bottles holding water doped with different concentrations of gadolinium‐diethylene triamine penta‐acetic acid (Gd‐DTPA), grocery store sunflower oil, and water were imaged together with the reference gel using the same $T_{1}$ and $T_{2}$ weighted scans mentioned previously.

### B. Comparison of images derived from RF head and body coils

The phantom was initially filled with materials commensurate with $T_{2}$ weighted imaging and scanned in a single acquisition using an appropriate $T_{2}$ weighted sequence (fast spin echo, TR/TE=4000/100 ms, image matrix=256×256, FOV=24×24 cm, BW=62.5 kHz, and 3 NEX). Similarly, the phantom was then imaged with a $T_{1}$ weighted sequence (spin echo, TR/TE=300/20, image matrix=256×256, FOV=24×24 cm, BW=16 kHZ, and 2 NEX). Region‐of‐interest analysis was carried out with mean and standard deviation of signal intensity values taken in fat, spine, brain, and background noise for both the $T_{1}$ and $T_{2}$ weighted scans in order to calculate contrast‐to‐noise ratio (CNR) and signal‐to‐noise ratio (SNR). Contrast‐to‐noise ratio is given by the following equation:

(1) $$\text{CNR}=\;\;\;\;(S_{1}-S_{2})/{\left(\sigma_{1}^{2}+\sigma_{2}^{2}\right)}^{1/2}$$

where $S_{1}$ and $S_{2}$ are signal intensities of two compartments of the phantom, and $\sigma_{1}$ and $\sigma_{2}$ are standard deviations of the same two compartments.

Signal‐to‐noise ratio is given by

(2) SNR=S/σ

where *S* is the signal intensity of the ROI, and σ is the standard deviation of the background

### C. Distortion of images with phantom scanned in body coil

#### C.1 Distortion

The presence of distortion in images of the phantom, acquired using the RF body coil of the scanner, was investigated. Flexible tubes filled with multimodality visible gel (Aqua Gel™, Adams Healthcare, Leeds, England) were connected to the periphery running parallel along the axis of the phantom in the 3, 6, 9, and 12 o'clock positions to facilitate accurate measurements of distance across the phantom. Magnetic resonance‐compatible spirit levels were attached to the front and end of the phantom in two planes to assist with alignment. A flat board insert was utilized to duplicate the treatment position, and three setup marks on the phantom were aligned with the land marking light on the scanner.

The phantom was imaged using a clinically relevant fast spin echo sequence (TR/TE=4000/100 ms, image matrix=256×256, FOV=28×28 cm, and 1 NEX). It is known^(1)^ ^,^ ^(16)^ that distortion effects are (inversely) proportional to receiver bandwidth. Hence, to investigate the degree of distortion under these acquisition conditions, four separate scans were acquired with receiver bandwidths of ±62.5,±31.0,±16.0, and ±9.0 kHz, respectively. The junction at the head and neck of the phantom was placed at the magnet isocenter, and 5 mm thick slices each separated by 5 mm were acquired, both inferior and superior to the isocenter, with the most distant slices 210 mm superior/inferior.

The positions of the horizontal and vertical markers were recorded from each axial image. Dimensions and positions were measured on image slices 0 (isocenter), 10, 20, 30, …, and 200 mm in the superior direction of the phantom.

#### C.2 Chemical Shift

The influence of chemical shift on the image geometry was examined. Images of the neck compartment of the phantom, which contains subcutaneous fat surrogate material, were examined from the sequence acquired above.

### D. Quantification of the accuracy of the registration of CT and MR images

#### D.1 Acquisition of images

For the acquisition of the CT images, the spinal cord of the phantom was filled with water doped with CT contrast (5.5%) in order to differentiate it from the bulk head–neck compartment. $T_{2}$ weighted MR images were obtained using a sequence optimized to minimize the distortion and chemical shift effects: spin echo, TE/TR=100 ms/4 s, 256×256 matrix, FOV=28×28 cm, BW=62.5 kHz, and 2 NEX. It was scanned in two acquisitions to obtain the head section and then the neck section. For each subset, the center of the imaging volume was positioned at the magnet isocenter and scanned 10 cm either side of this point.

Both the CT and MR image sets were obtained as 5 mm thick contiguous slices. The phantom was aligned in both scanners using patient setup orientation marks and sagittal and lateral side crosses, and was placed on a board to ensure exact positional reproducibility and consistency during an MR or CT image acquisition. Furthermore, MR‐compatible spirit levels attached to the phantom helped ensure it was level at setup.

In addition, we acquired MR images with the phantom deliberately misaligned to test the registration software's capability and accuracy. These were as follows:

(1) with the front of the phantom (base of neck) lifted by 1 cm
(2) with the right‐hand side of the phantom lifted by 1 cm
(3) with the phantom rotated about the front “left” corner, so that the back left corner was translated by 1 cm

#### D.2 Image registration and “organ” delineation

The accuracy of the registration process between head and neck CT and MR images was investigated. We are using the FOCALFusion image registration software from CMS with the CT images, the primary data set, to which the MR images, the secondary data set, are registered. During the registration, using the mutual information algorithm, a transformation is found to align the secondary data set to the primary set. This process results in the secondary set being interpolated and reformatted to have the same slice location and resolution as the primary set. Once registered, the two data sets can be used interchangeably. All structures were contoured on CT, blind of the MR images and vice versa. The contours from the two modalities can be qualitatively compared.

#### D.3 Quantitative comparison

The contour data was extracted from ASCII files using in‐house software. The contours for the trachea, inner neck and fat, and spine and head were recorded at representative axial slice positions throughout the phantom. The “center‐of‐mass” of evenly distributed points placed around the original noncontiguous pixel boundary was also determined using in‐house software. In addition, the area and perimeter were also calculated, and the following coefficient of agreement (*A*) was determined:

(3) $$A=\sqrt{R_{a}R_{p}}$$

where $R_{a}$ is the ratio of CT area to MR area, and $R_{p}$ is the ratio of CT perimeter to MR perimeter for a given contour. To test the sensitivity of this measurement, we compared a square to a circle and a triangle to a circle. Comparison of a square and circle with the same area and center results in A=1.06. Comparison of a triangle and circle, where the vertices of the triangle lie on the circle, results in A=1.71.

## III. RESULTS

### A. Head and Neck Phantom

Pixel intensity values varied least in the gel, with the SD approximately 6% of the mean value. There was more variability in the volunteer ROIs, giving a SD of 26% and 14% for the $T_{1}$ and $T_{2}$ weighted scans, respectively. Mean±SD values for the $T_{1}$ ratios were 1.37±0.16,0.54±0.02, and 0.19±0.01 for fat, brain, and CSF, respectively, and corresponding values for $T_{2}$ ratios were 0.82±0.04, 0.53±0.05, and 1.19±0.08.

The results in the various materials were as follows. The $T_{1}$ ratio for undoped water was 0.30. Sunflower oil produced ratios of 1.43 and 0.73 for $T_{1}$ and $T_{2}$ sequences, respectively. From a plot of $T_{1}$ and $T_{2}$ ratios against gadolinium concentration, the appropriate brain and CSF compartments of the phantom were chosen. The $T_{1}$ phantom, therefore, consisted of water with 0.1 mL/L Gd‐DTPA concentration for brain/background contrast, undoped water for spinal cord contrast, and sunflower oil for subcutaneous fat contrast. The $T_{2}$ phantom consisted of water plus 3.5 mL/L GD‐DTPA for brain/background contrast, water plus 2.0 mL/L GD‐DTPA for spinal cord contrast, and sunflower oil for subcutaneous fat contrast. In each case, sunflower oil was chosen rather than suitably doped water both to mimic signal intensity and to provide an appropriate chemical composition.

### B. Comparison of head and body coil

Values for SNR and CNR obtained with each coil and for both the $T_{1}$ and $T_{2}$ weighted scans are shown in Tables I(a) and I(b).

**Table I(a):** Head and body coil comparison results for $T_{1}$ weighted sequence

Phantom compartment	Body coil CNR	Head coil CNR	Body coil SNR	Head coil SNR
spine	9.0	15.5	19.9	53.4
fat	9.7	10.9	37.7	188.8
head	10.2	15.7	13.7	66.5

**Table I(b):** Head and body coil comparison results for $T_{2}$ weighted sequence

Phantom compartment	Body coil CNR	Head coil CNR	Body coil SNR	Head coil SNR
spine	13.3	14.4	40.9	157.9
fat	7.2	10.3	22.0	92.0
head	13.1	14.2	30.8	119.7

SNR obtained with the head coil is much greater than with the body coil, with a mean ratio (head SNR/body SNR) of 4.1 for $T_{1}$ sequences and 3.9 for $T_{2}$ sequences. However, the CNR values are similar, with a mean ratio (head CNR/body CNR) of 1.4 for $T_{1}$ sequences and 1.2 for $T_{2}$ sequences.

### C. Distortion and Chemical Shift

#### C.1 Distortion

**Figure 2:**
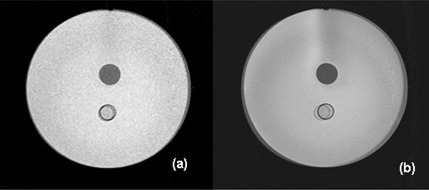
This two axial images obtained using a $T_{2}$ weighted sequence. The image acquired at the isocenter of the magnet is the darker or less transparent of the two images; overlaid on it is that obtained at 200 mm superiorly from the isocenter. was obtained using a bandwidth of 62.5 kHz, while a value of 9 kHz was used for The small bandwidth image clearly demonstrates the greatest distortion.

Figs. 2(a) and (b) show an axial $T_{2}$ weighted image acquired at a distance of 200 mm from the magnet isocenter, together with an image acquired at the isocenter superimposed (darker image).

Fig. 2(a) displays a slight shift in image position of the 200 mm image compared with the corresponding image at the isocenter using a 62.5 kHz bandwidth. The same image comparison at 9 kHz (Fig. 2(b) displays a much greater disparity and implies that image distortion is responsible. In both cases, images at each slice position were viewed in a cine format, and the shift was observed to be minimal up to 100 mm from the isocenter and then deviated noticeably with the maximum effect at 200 mm distance. Therefore, these shifts could not be simply a misalignment of the phantom itself.

**Figure 3:**
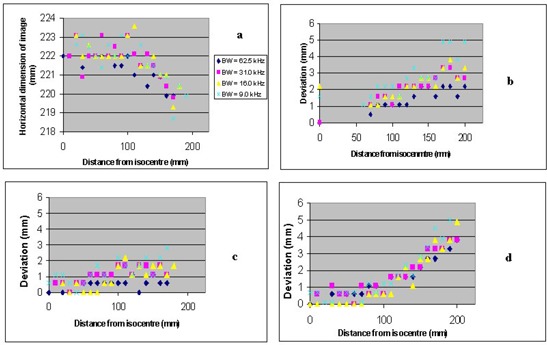
Plots showing (a) horizontal image dimensions, (b) position of anterior marker, (c) position of right marker, and (d) position of left marker relative to the gold standard image (described in text). It should be noted that all shifts seen in (b) to –(d) are to the right (taken as positive in our convention). Some data points are missing [e.g., 10–50 mm in (b)] because the marker tubes were not uniformly filled with gel.

The results of horizontal dimension measurements and image shift relative to the image at the isocenter $\left(S_{0}\right)$ for bandwidth 62.5 kHz (taken as gold standard image) are shown in Figs. 3(a) to (d).

The precision with which the marker tubes were attached to the phantom together with the nonuniformity of the filling may be responsible for the apparent noise in the plots. Fig. 3(a) indicates that as the phantom is scanned farther away from the isocenter, the horizontal dimension begins to shrink, with smaller bandwidths exhibiting the largest change. This finding is consistent with the image in Fig. 2(b). However, this shrinking effect only seems prominent beyond 150 mm from the isocenter. Bandwidths 62.5, 31.0, 16.0, and 9.0 kHz yielded shrinkages (isocenter image compared to 200 mm superior) of 2.1, 2.2, 2.7, and 2.7 mm, respectively.

Figs. 3(a) to (d) illustrate a shift of the phantom images (when normalized to the gold standard image) to the right, which is again consistent with the image in Fig. 2(b). These shifts are more prominent at a distance greater than 150 mm from the isocenter, with smaller bandwidths demonstrating greater deviations.

#### C.2 Chemical Shift

**Figure 4:**
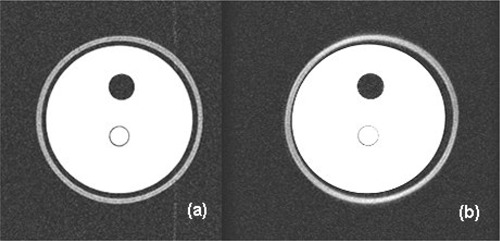
Image of neck compartment of phantom, the bulk of the filling of which is (doped) water whereas the outer rim contains a fat surrogate. Two receiver bandwidths were used: (a) 62.5 kHz and (b) 9 kHz. The latter clearly shows a distortion due to the chemical shift effect.

Fig. 4(b) shows an artifactual shift in the outer “fat” layer in the direction of the frequency encoding gradient (left‐right). This image was acquired with a 9 kHz receiver bandwidth, whereas this effect is not seen in Fig. 4(a) (acquired with a bandwidth of 62.5 kHz). This result was found to be independent of distance from the isocenter and is due to the chemical shift difference of the proton resonance in fat and water.

Using knowledge of the image matrix size and receiver bandwidth, a theoretical calculation of the magnitude of this effect can be determined. The chemical shift of fat and water at 1.5 T is approximately 225 Hz. This is divided by the finite frequency range, which represents each pixel (given by bandwidth/matrix in hertz/pixel). Using a bandwidth of 62.5 kHz yields a shift of 0.46 pixels, and a bandwidth of 9.0 kHz corresponds to a shift of 3.2 pixels. At the nominal scan resolution, the latter figure equates to a 3 mm separation. This is consistent with observations; the fat appears to almost “bridge” the Perspex gap in the neck compartment.

### D. Image registration

#### D.1 Analysis of registration between “aligned image sets”

In the case where the phantom was aligned perfectly in the MR scan acquisition, the maximum difference between the *x* center of shape coordinates was, Δx(max)=1.30 mm and (the mean±SD) Δx (mean)=0.43±0.14 mm. The maximum difference between the *y* center of shape coordinates was Δy (mean)=1.00 mm and Δy (mean)=0.37±0.07 mm. The coefficients of agreements on each image slice were close to unity, with the maximum value $A_{\text{max}}$ of 1.027 and the mean value $A_{\text{mean}}$ of 1.004. This indicates good agreement between CT and MR area values and between CT and MR perimeter values.

#### D.2 Analysis of registration with front of phantom (base of neck) lifted by 1 cm during the MR scan

The values obtained from this scan were the following: $\Delta x\;(\text{max})=1.40\;\text{mm},\;\Delta x(\text{mean})=0.59\pm 0.28\;\text{mm},\;\Delta y(\max)=-1.40\;\text{mm},\;\Delta y(\text{mean})=0.39\pm 0.26\;\text{mm},A_{\max}=1.093$, and $A_{\text{mean}}=1.014$.

#### D.3 Analysis of registration with the right‐hand side of the phantom lifted by 1 cm during the MR scan

Corresponding values are the following: $\Delta x(\max)=0.60\;\text{mm},\Delta x(\text{mean})=0.29\pm 0.07\;\text{mm},\;\Delta y(\max)=1.80\;\text{mm},\;\Delta y(\text{mean})=0.58\pm 0.52\;\text{mm},A_{\max}=1.025$, and $A_{\text{mean}}=0.998$.

#### D.4 Analysis of registration with the phantom rotated/pivoted about the front left corner of the phantom, so that the back left corner was translated by 1 cm during the MR scan

Similarly, values were as follows: $\Delta x(\max)=-160\;\text{mm},\Delta x(\text{mean})=0.45\pm 0.18\;\text{mm},\Delta y(\max)=0.90\;\text{mm},\Delta y(\text{mean})=0.29\pm 0.05\;\text{mm},A_{\max}=1.055$, and $A_{\text{mean}}=1.010$.

### IV. DISCUSSION

Using the materials described in this paper we have constructed a quality assurance phantom to evaluate MR distortions and registration accuracy. In addition, the phantom may be used to investigate various imaging sequences and RF coils.

Signal‐to‐noise is on average four times greater for images acquired in the head coil relative to the body coil. However, contrast‐to‐noise is approximately of the same magnitude between head and body coils for both $T_{1}$ and $T_{2}$ weighted scan sequences, which suggests that head and neck patients can be imaged in the body coil without compromising the differentiation of anatomical features.

In MRI, linear changes in magnetic field are used to spatially encode the final image. Any perturbations to this linearity will produce image distortions. Inhomogeneities in the main magnetic field or gradients will therefore have a detrimental effect on the final image. While the inhomogeneity of the main field is kept to a minimum under stringent manufacturer specifications, previous studies have demonstrated distortions of several millimeters,^(16)^ with the greatest effect observed at the periphery. For open magnets the problem is considerably worse, and 15 mm deviations have been recorded.^(17)^ Our results, using a 1.5 T system, have shown that while distortion effects are present, they can be minimized by an appreciation of the receiver bandwidth (gradient strength) and distance from the isocenter ($B_{0}$ homogeneity). In consideration of both chemical shift and distortion, images should be acquired with high receiver bandwidths, preferably ≥ 30 kHz, and should not extend beyond 15 cm from the isocenter.

There is very good agreement between CT and MR area values and between CT and MR perimeter values. Our analysis showed that a maximum coefficient of agreement is only 9% greater than unity and the maximum mean value only 2.7% above unity. In section II D.3 we showed that comparison of contours that were very similar (circle and square of equal area and center) scored a coefficient of 6% greater than unity. There was no significant deviation from these results for the misaligned scans, suggesting that the mutual information registration algorithm used in the FOCALFusion software can manage with offsets up to 1 cm. The mean value for the measured coefficients of agreement was equal to 1.028, which suggests a small but systematic overestimate of contour shapes/sizes drawn on the CT images. The largest difference in any center of shape coordinate measurement was 1.8 mm. When mean values of this parameter for each region were examined, the largest value was 0.45 mm. Corresponding values from the misaligned scans were 0.43, 0.59, and 0.58 mm. These values are easily within the tolerances for alignment and contouring.

We have shown how a simple phantom can be used to characterize the registration process when utilizing two different imaging modalities. It should be understood, however, that there are three main contributors in such a process, the third being the software used for registration. It is important that the final fused image data sets are compared and analyzed when performing quality assurance on the use of multimodality fusion for treatment planning of radiotherapy.

Furthermore, we have shown that some image distortion is seen in axial images, even when using acquisition sequences designed to minimize it. Having understood the practical limitations, scanning protocols can be formulated that really negate the impact of any such (small) effects when using MR imaging to delineate the tumor and proximal tissues. However, it is important to recognize that each MR scanner will perform differently, and systematic analysis of these factors should be performed as part of quality assurance to ensure the accuracy of the planning procedure.

Finally, we believe that our work shows that MR imaging can be safely used in treatment planning of radiotherapy for the head and neck; given its enhanced soft tissue delineation, it will provide an excellent tool for more discriminate contouring as we implement novel radiotherapy methods, such as intensity‐modulated radiotherapy.^(18)^ The work also shows that “discrepancies” seen in perceived structure or tumor delineation in soft tissues can be assumed to be due to the imaging capabilities of the media in question rather than phantom effects from the registration process itself.

We are currently extending this work and are redesigning the phantom, making it possible to internally mount flasks containing a gel dosimeter^(9)^ ^,^ ^(19)^ in order to make a universal phantom for head and neck radiotherapy. The addition of this facility will allow us to use the phantom for planning highly conformal treatments and then verify the delivery (using gel dosimetry) to validate the *entire* process.

## ACKNOWLEDGMENTS

We are grateful for the financial support of Yorkshire Cancer Research and Hull & East Hospitals NHS Trust. We are also thankful to Computerized Medical Systems for their interest and the assistance provided for our work. The authors would also like to thank Rob Walker and his staff in the medical physics workshop at Princess Royal Hospital for the construction of the phantom.
